# Effects of Non-Thermal Plasma on Mammalian Cells

**DOI:** 10.1371/journal.pone.0016270

**Published:** 2011-01-21

**Authors:** Sameer Kalghatgi, Crystal M. Kelly, Ekaterina Cerchar, Behzad Torabi, Oleg Alekseev, Alexander Fridman, Gary Friedman, Jane Azizkhan-Clifford

**Affiliations:** 1 Department of Electrical and Computer Engineering, Drexel University, Philadelphia, Pennsylvania, United States of America; 2 Department of Biochemistry and Molecular Biology, Drexel University College of Medicine, Philadelphia, Pennsylvania, United States of America; 3 Department of Surgery, Drexel University College of Medicine, Philadelphia, Pennsylvania, United States of America; 4 Department of Mechanical Engineering and Mechanics, Drexel University, Philadelphia, Pennsylvania, United States of America; Massachusetts Institute of Technology, United States of America

## Abstract

Thermal plasmas and lasers have been widely used in medicine to cut, ablate and cauterize tissues through heating; in contrast, non-thermal plasma produces no heat, so its effects can be selective. In order to exploit the potential for clinical applications, including wound healing, sterilization, blood coagulation, and cancer treatment, a mechanistic understanding of the interaction of non-thermal plasma with living tissues is required. Using mammalian cells in culture, it is shown here that non-thermal plasma created by dielectric barrier discharge (DBD) has dose-dependent effects that range from increasing cell proliferation to inducing apoptosis. It is also shown that these effects are primarily due to formation of intracellular reactive oxygen species (ROS). We have utilized γ-H2AX to detect DNA damage induced by non-thermal plasma and found that it is initiated by production of active neutral species that most likely induce formation of organic peroxides in cell medium. Phosphorylation of H2AX following non-thermal plasma treatment is ATR dependent and ATM independent, suggesting that plasma treatment may lead to replication arrest or formation of single-stranded DNA breaks; however, plasma does not lead to formation of bulky adducts/thymine dimers.

## Introduction

The term plasma in physics refers to a partially ionized medium, usually gas. Importantly, plasma not only produces electrons and various ions, but also neutral (uncharged) atoms and molecules, such as free radicals and electronically excited atoms having high chemical reactivity and the capability to emit UV. The temperature and components of the gas, as well as the strength and pulse duration of the electric field determine the exact composition of plasma. In man-made systems, plasma is usually generated by electrical discharges and can be generally classified according to its gas temperature. In thermal plasma, gas temperature can reach several thousand degrees Kelvin. Devices, such as argon plasma coagulators, which are used clinically to cauterize living tissues, typically generate plasmas at temperatures far exceeding room temperature. The effects of such thermal plasmas on tissues are non-selective and difficult to control because they occur primarily through transfer of intense heat [1]. In contrast, in non-thermal plasmas, gas can be maintained close to room temperature. Although electrical discharges that generate non-thermal plasma have been known for a long time, their clinical potential has been largely ignored and until recently, applications have been confined to sterilization of inert surfaces [2], [3], [4], [5], [6], [7], [8], [9], [10], [11] or modulation of cell attachment [12], [13] through surface modification. It has recently been demonstrated that non-thermal atmospheric pressure plasma can be applied directly to living cells and tissues [11], killing bacteria and inducing blood coagulation without significant heating [11], [14]. Non-thermal plasma treatment has also been shown to promote cell proliferation [15], enhance cell transfection [16], [17], sterilize root canals [18], [19], [20] and possibly increase wound healing [21]. The simplicity and flexibility of devices required to generate non-thermal plasma and apply it to tissues is particularly appealing. However, an understanding of mechanisms by which non-thermal plasma interacts with living cells and tissues is required to fully develop its clinical applications.

**Figure 1:**
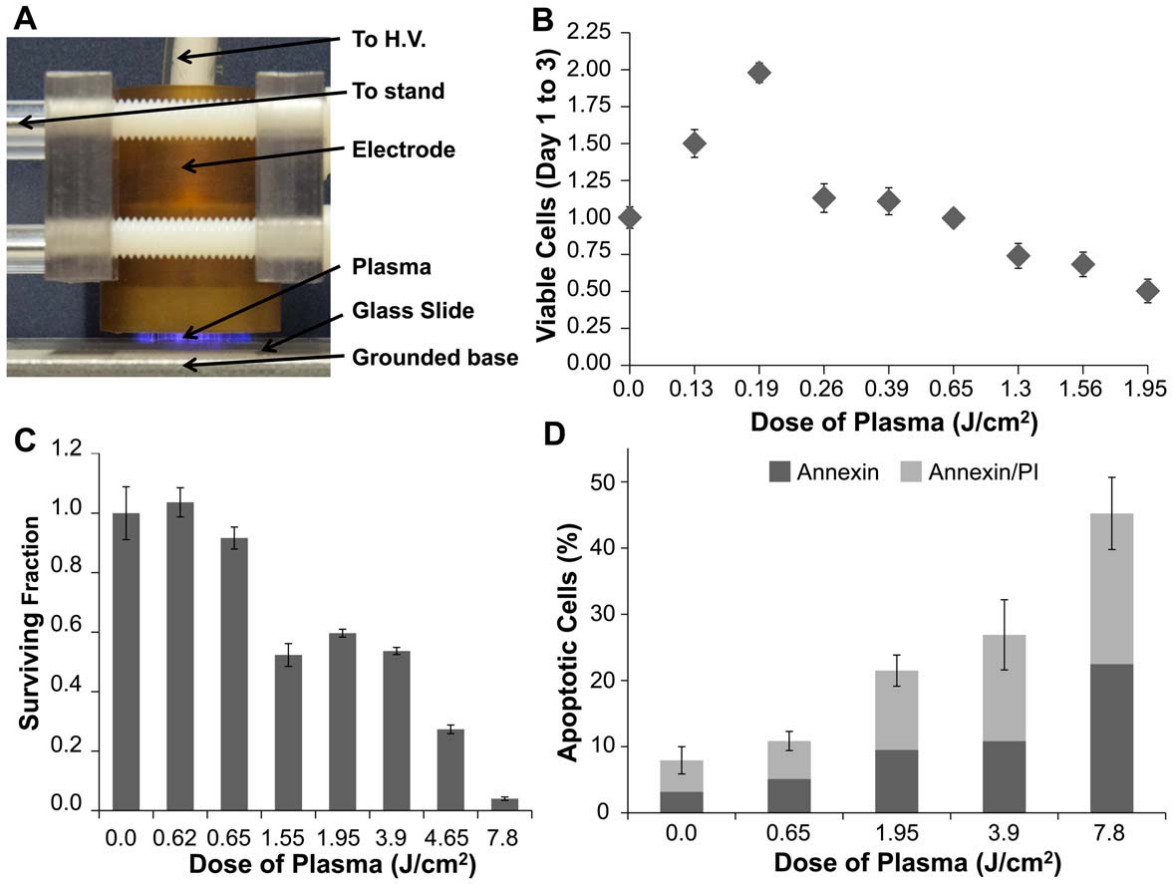
Dose-dependent effects of non-thermal atmospheric pressure dielectric barrier discharge (DBD) plasma on MCF10A cells. (**A**) Photograph of DBD plasma treatment of cells. (**B**) 10^4^ MCF10A cells plated on glass cover slips were treated with the indicated dose of DBD plasma as described. Cells were counted 24 and 72 hours after treatment. Data are plotted relative to the # of cells in the untreated plate at 24 hours relative to the # at 72 hours, which was set at 1.0. (**C**) Cells were treated with the indicated dose of DBD plasma; and colony survival assays were performed as described. Data are expressed relative to the # of colonies in the untreated control. (**D**) Three days after treatment with the indicated dose of DBD plasma, cells were harvested and stained with Annexin V/propidium iodide (PI) and analyzed by Guava.

Several different methods of non-thermal plasma generation at atmospheric pressure are known [22]. The type of non-thermal plasma employed in this study is called Dielectric Barrier Discharge (DBD) [23], which occurs at atmospheric pressure in air when high voltage of time-varying waveform is applied between two electrodes, with at least one electrode being insulated [24], that prevents current build-up, creating electrically safe plasma without substantial gas heating (Figure 1.). This approach allows direct treatment living tissues without thermal damage [1]. Plasma is an ionized gas composed of charged particles (electrons, ions), electronically excited atoms and molecules, radicals, and UV photons. Plasma treatment exposes cells or tissue surface to active short and long lived neutral atoms and molecules, including ozone (O_3_), NO, OH radicals, and singlet oxygen (O_2_^1^Δ_g_), and a significant flux of charged particles, including both electrons and positive and negative ions like super oxide radicals [22], [25], [26]. Non-thermal plasma density, temperature, and composition can be changed to control plasma products.

Prior studies have focused mainly on bactericidal effects of plasma [27], which require the presence of oxygen [10], [28], consistent with the suggestions in the literature that oxidative stress (among other factors) may be mediating the interaction between non-thermal plasma and living organisms [4], [5], [13]; however, to date there are no data to indicate that plasma treatment induces oxidative stress in cells. Since plasma does not produce sufficiently energetic particles or photons to penetrate cells, it had been assumed that cellular genetic material would not be affected; however, formation of intracellular ROS is the major mechanism by which ionizing radiation produces DNA damage [29]. DNA damage resulting from ROS includes small or bulky modifications to bases, interstrand and intrastrand cross-links, as well as single-strand and double-strand breaks (DSB) [30]. DNA damage induces a cascade of signals through activation of cellular kinases, including the phosphatidylinositol 3-kinase–related kinases (PIKK), specifically ataxia-telangiectasia mutated (ATM), DNA dependent protein kinase (DNA-PK), and ATM and Rad3-related (ATR), which phosphorylate the histone variant H2AX in the vicinity of damage [31]. We demonstrate that non-thermal plasma induces a variety of effects on mammalian cells, ranging from increased cell proliferation to apoptosis, and it leads to DNA damage through formation of intracellular ROS. The DNA damage signaling cascade activated by non-thermal plasma treatment is different from that associated with ionizing radiation (IR), ultraviolet (UV) or H_2_O_2_ [29].

## Materials and Methods

### Cell Culture

Mammalian breast epithelial cells (MCF10A) were maintained in high glucose Dulbecco's Modified Eagle's Medium/Ham's F12 50∶50 mixture (Cellgro, Mediatech, VA, USA) supplemented with 5% horse serum, Epidermal Growth Factor (100 µg/ml), Hydrocortisone (1 mg/ml), Cholera Toxin (1 mg/ml), Insulin (10 mg/ml) and Penicillin/Streptomycin (500 µl, 10000 U/ml penicillin and 10 mg/ml streptomycin), which were all purchased from Sigma, St Louis, MO. For plasma treatment, cells were washed with phosphate buffered saline (PBS), detached with 0.25% Trypsin (GIBCO, Invitrogen, CA, USA), and seeded near confluence (4×10^5^ cells/well) on 22×22 mm square glass cover slips (VWR, PA, USA) in 6-well plates (Greiner Bio One, NC, USA). Cells were cultured in complete medium in a 37°C, 5% CO_2_ incubator for 24 hours prior to plasma treatment.

Amino acids serine, methionine, cysteine, arginine, leucine, lysine, isoleucine, valine, proline, glutamate and glutamine (100 µM, Sigma, St Louis, MO) were used to treat cells directly and separately. N-Acetyl L-cysteine (NAC, 4 mM, Sigma, St Louis, MO), was used as an intracellular reactive oxygen species scavenger.

### Plasma Treatment

DBD plasma was produced using an experimental setup shown in Figure 1A and schematically illustrated in Figure S1 [11]. Plasma was generated by applying alternating polarity pulsed (500 Hz –1.5 kHz) voltage of 20 kV magnitude (peak to peak), 1.65 µs pulse width and a rise time of 5 V/ns between the high voltage electrodes using a variable voltage and variable frequency power supply (Quinta, Russia). One mm thick quartz glass was used as an insulating dielectric barrier covering the 1-inch diameter copper electrode. The discharge gap between the bottom of the quartz and the treated sample surface was fixed at 2 mm. Discharge power density was measured to be 0.13 Watts/cm^2^ (at 500 Hz) and 0.31 Watts/cm^2^ (at 1.5 kHz) [32]. Plasma treatment dose in J/cm^2^ was calculated by multiplying the plasma discharge power density by the plasma treatment duration. For example, plasma treatment at a power density of 0.13 W/cm^2^ for 15 s would correspond to a dose of 1.95 J/cm^2^. Non-thermal DBD plasma produces various ROS in gas phase whose typical concentrations are provided in Table 1 [22], [25], [33]. The dependence of ROS concentration on plasma power density is complex [24], [34]. DBD plasma has a g-factor (number of ROS generated per electron volt or eV) between 0.3 and 0.5 [35]. For a plasma dose of 3.9 Joules/cm^2^, about 5×10^16^ ROS are generated. Characterization of DBD plasma employed here has been presented elsewhere [11], [32]. Importantly, ozone (O_3_) concentration of about 100 ppm obtained at typical operating conditions employed here was much higher than 1-ppm level concentration of nitric oxide (NO), which is entirely consistent with other published reports [4], [5], [34]. Concentration of ozone produced by DBD plasma in the gas phase was measured using an optical ozone meter MedOzon-245/5 (MedOzone, Russia) and the concentration of NO was measured using a NO/NOx chemiluminescent analyzer Model 600 CLD (California Analytical Instruments, USA).

**Table 1:** Typical relative concentrations of various charged and neutral species generated by non-thermal DBD plasma in gas phase [25], [33], [41].

Plasma Generated Species	Density (cm^−3^)
**Superoxide (O_2_•^−^)**	10^10^–10^12^
**Hydroxyl (OH•)**	10^15^–10^17^
**Hydrogen Peroxide (H_2_O_2_)**	10^14^–10^16^
**Singlet Oxygen (^1^O_2_)**	10^14^–10^16^
**Ozone (O_3_)**	10^15^–10^17^
**Nitric Oxide (NO)**	10^13^–10^14^
**Electrons (e^−^)**	10^9^–10^11^
**Positive Ions (M^+^)**	10^10^–10^12^

MCF10A cells on glass cover slips were exposed to plasma at various doses (0.13–7.8 J/cm^2^). Briefly, each cover slip was removed from the 6-well plate, drained, and placed on a microscope slide. 100 µl of complete medium or defined medium was added to the glass cover slip before plasma treatment to prevent sample drying. After treatment, cells were held in the treated medium for one minute (unless indicated otherwise) and then the cover slip was placed in a new 6-well plate containing 2 ml of complete medium, and the samples were returned to the incubator for one hour before analysis by immunofluorescence or western blot.

In some experiments, medium (complete or defined) or PBS was treated separately from the cells, designated ‘separated treatment’. In these cases, 100 µl of medium was placed on a cover slip and treated with plasma as described for the cells. The treated medium was then transferred to a cover slip on which cells had been plated as described above.

During plasma treatment with or without cells present, the surface of the medium is exposed to plasma and charged species (electrons and ions) and uncharged gas species [including some with relatively short half-life (OH, O, for example)] reach the surface. A grounded mesh was placed between the high voltage copper electrode and the surface of the medium to block electrons and ions and allow only uncharged gas species to reach the medium surface. Similarly, a magnesium fluoride (MgF_2_) glass which is practically transparent to UV, but not to any gas species, charged or uncharged, was placed over the top of the medium surface to test for possible effects of UV.

### Cell Growth Assay

MCF10A cell proliferation was measured through cell counts on directly treated cells. MCF10A cells (1×10^4^) were seeded on 22×22 mm cover slips in 6-well plates one day before plasma treatment. Cells were plasma-treated as described and incubated for an additional 3 days with a medium change on day 2. Cell number was quantified on days 1 and 3 by counting trypsin-detached cells using a Cell Viability Assay (Guava EasyCyte Plus, Millipore, MA, USA). Fold growth was determined by taking the ratio of the number of attached cells on day three to day one.

### Colony Survival Assay

MCF10A cells (4×10^5^) were seeded on 22×22 mm cover slips in 6-well plates one day before plasma treatment. One day after treatment with plasma or H_2_O_2_ (positive control), 300 cells were seeded onto 60-mm dishes. Eleven days after plating, cells were fixed and stained with a crystal violet solution (0.5% in 20% ethanol), and colonies were counted.

### Apoptosis

Apoptosis was measured via Annexin V/propidium iodide labeling. Annexin V binds phosphatidylserine translocated from the inner to the outer cell membrane. Cells in early apoptosis are identified as Annexin V-positive and negative for the vital dye propidium iodide. Floating and trypsin-released cells were collected and centrifuged, washed thoroughly, resuspended in Annexin binding buffer, and labeled with Annexin V-fluorescein and propidium iodide as per manufacturer instructions (BD Pharmingen, San Jose, CA). Samples were analyzed immediately by flow cytometry (Guava EasyCyte Plus, Millipore, MA, USA).

### Immunofluorescence

MCF10A cells were plated 24 h before treatment. One hour after plasma treatment, cells were subjected to *in situ* cell fractionation as described [36], by incubation in pre-extraction buffer (1X PBS +0.2% Triton-X +1∶50 PMSF) for 5 min at 4C, followed by one wash with PBS and incubation in fixation solution (3% paraformaldehyde +2% Sucrose in PBS) for 10 min at room temperature. Cells were then washed in PBS and incubated in permeabilization buffer (1X PBS +0.5% Triton-X) for 5 min a 4°C. Cells were washed twice with NaN_3_+ PBST at room temperature and incubated overnight at 4C in primary antibody (mouse monoclonal γ-H2AX antibody, serine 139, Upstate Biotechnology, 1∶1000). After three washes in NaN3+ PBS, cells were incubated for 1 h in the dark in secondary antibody (AlexaFlour594 donkey anti-mouse antibody, diluted 1∶1,000) followed by incubation of slides in 1 µl DAPI + PBST + NaN_3,_ three washes in NaN_3_ + PBST and mounting using DAPI-free mounting medium (Vector Labs) on glass microscope slides overnight. The slides were then frozen at −20°C for one day prior to imaging them on an upright fluorescence enabled microscope.

### Western Blot

Protein expression and modification were analyzed by immunoblot. Total cell lysates were prepared by direct lysis of washed cells in 2X SDS sample buffer containing 5% β-mercaptoethanol. Samples were electrophoresed at 150 V in Tris-glycine SDS running buffer (25 mmol/L Tris, 192 mmol/L glycine, 0.1% SDS (pH 8.3)). Following electrophoresis, proteins were transferred to PVDF membrane ((Millipore, MA, USA) for two hours in Tris-glycine transfer buffer (10% SDS, Deionized Water, Tris-Glycine and Methanol (VWR, PA USA)). Immunoblotting was done by blocking membranes in 1% nonfat dried milk (Carnation) in PBS with 0.1% Tween 20 (PBST) for α-tubulin or 5% bovine serum albumin (BSA, Fraction V, Fisher Scientific) in PBST for γ-H2AX, followed by incubation with primary antibodies overnight for 10 to 12 h at 4°C with rocking. Primary antibodies used for immunoblot included mouse monoclonal antibodies specific for γ-H2AX [phospho-histone H2AX (serine 139), clone JBW301; Upstate] and α-tubulin (Santa Cruz Biotechnology). The primary antibodies were detected with fluorescently tagged goat anti-mouse Alexa and Fluor 488 (Santa Cruz Biotechnology). Immunoblot was developed using Odyssey Infrared Gel Imaging system (LI-COR Biosciences, NE, USA).

### Detection of Intracellular Reactive Oxygen Species

The intracellular generation of reactive oxygen species after plasma treatment was detected using the fluorescent probe 5-(and-6)-chlroromethyl-2′, 7′-dichlorodihydrofluorescein diacetate, acetyl ester (CM-H_2_DCFDA, Molecular Probes). Untreated cells, cells treated with 100 µM H_2_O_2_ and plasma treated cells were analyzed for changes in fluorescence. MCF10A cells (4×10^5^) were plated one day before plasma treatment. On the day of the experiment, the cells were washed 2X with PBS, and then preincubated with 10 µM CM-H_2_DCFDA at 37°C for 30 min in the dark. After 30 min, the excess dye was washed off with PBS and cells were incubated in complete medium at 37°C in the dark for 30 min, during which time, the acetate groups on CM-H_2_DCFDA are removed by intracellular esterases, trapping the probe inside the cells. Production of ROS was measured by detecting changes in the fluorescence of dichlorofluorescein 5, 30, and 60 min after plasma treatment.

### Lentivirus Production and Cell Transduction

Lentivirus was prepared from MISSION shRNA (Sigma Aldrich, St Louis, MO, USA) following the manufacturer's instructions for targeting ATM (NM_000051.2-9380; NM_000051.2-2990) and ATR (NM_001184.2-231); pLKO-Non-targeting (shC002) and pLKO-GFP (shC104) were used as controls. The shRNA plasmids were then transfected into 293T cells with VSVG, RRE and RSV-Rev packaging vectors to generate corresponding pseudoviruses. Virus was collected 48 h post transfection. For stable knockdown, 72 h after transduction, MCF10A cells were selected in 1 µg/ml puromycin for 48 h.

### Formation of Thymine Dimers

The published protocols [37], [38] were used to measure formation of thymine dimers after being modified as follows. Monolayers were rinsed twice with PBS and then fixed with 3% paraformaldehyde in washing buffer (WB: 0.1% Triton X-100 in PBS) for 20 min on ice. Cells were rinsed twice in PBS, permeabilized with WB for 15 min on ice, and rinsed two more times with PBS. DNA was denatured with 2 N HCl for 5 min at 37°C, and the acid was neutralized by washing five times with PBS. Non-specific binding was blocked with 20% BSA in WB for 30 min at 37°C while shaking. Cells were incubated with the TDM-2 primary antibody (Kindly provided by Dr. Toshio Mori at the Nara Medical University, Nara, Japan) against cyclobutane pyrimidine dimers at a 1∶150 dilution in 1% BSA in WB for 1 h at 37°C. After five rinses with 1% BSA in WB, cells were incubated with the Alexa Fluor 594 anti-mouse secondary antibody (Invitrogen, Carlsbad, CA) at a 1∶400 dilution in 1% BSA in WB for 30 min at 37°C. Nuclei were counterstained with 10 µg/ml Hoechst 33258 for 15 min at 37°C. Cells were rinsed five times with PBS before mounting with Vectashield mounting medium (Vector Laboratories, Burlingame, CA). All slides were visualized with Olympus AX70 compound epifluorescence microscope equipped with Spot RT Slider camera.

### Statistical analysis

All experimental data points were from triplicate samples and are expressed and/or plotted as the mean ± S.E.M. Data were analyzed by Student's t-test to establish significance between data points.

## Results

In order to test the effects of plasma treatment on mammalian cells, DBD plasma was applied to human breast epithelial (MCF10A) cells (Figure 1A and Figure S1). Plasma is delivered from a probe placed at a fixed distance over the media being treated; plasma is released as an electrical discharge as a result of electrical breakdown of the air in the gap between the high voltage electrode and the substrate being treated. The initial experiment involved establishing the dose-dependent effects of plasma treatment on cell proliferation and survival by direct cell count and colony formation. At low doses, the rate of cell proliferation increased; the cell number between days 1 and 3 in cells treated at 0.19 J/cm^2^ was twice that in the untreated control, and at intermediate doses (0.26–0.65 J/cm^2^), there was no significant effect on proliferation as compared to untreated cells (Figure 1B). At doses between 1.3 and 1.95 J/cm^2^, cell number decreased. There was some correlation between growth inhibition and survival, at a dose of 1.55 or 3.9 J/cm^2^—survival was inhibited by about 40% (Figure 1C). Annexin V/PI staining of cells treated with plasma at doses ranging from 0.65 to 3.9 J/cm^2^ revealed that apoptosis was induced at higher doses (1.95 J/cm^2^ and higher) where decreased survival was also observed (Figure 1D).

**Figure 2:**
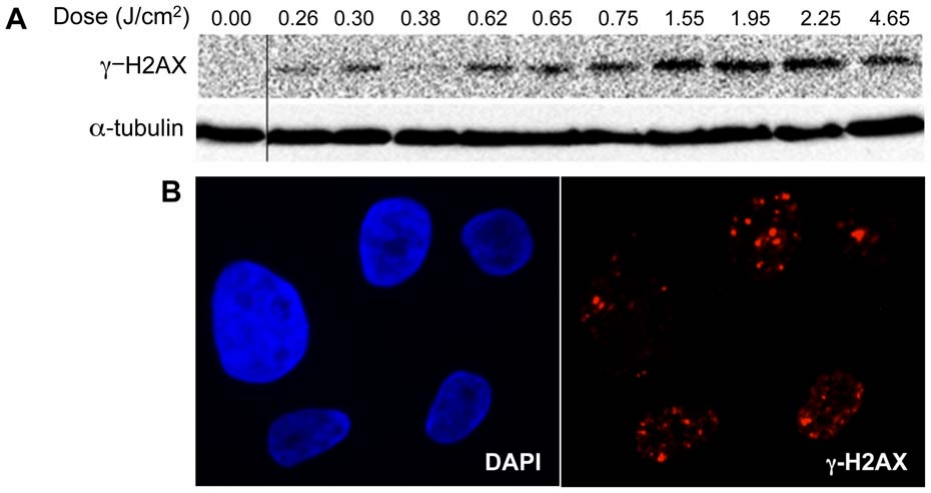
Induction of DNA damage by DBD plasma. (**A**) MCF10A cells were treated with the indicated dose of plasma. After one-hour incubation, lysates were prepared and resolved by SDS-PAGE and representative immunoblots with antibody to γ-H2AX (top) or α-tubulin (bottom) are shown. (**B**) Indirect immunofluorescence was performed utilizing an antibody to γ-H2AX one hour after treatment of MCF10A cells with 1.55 J/cm^2^ DBD plasma.

One possible mechanism underlying these dose-dependent effects is generation of intracellular ROS, which at low levels is known to increase cell proliferation and at high levels induces cell death through DNA damage [30]. To determine whether DBD plasma treatment of cells could induce DNA damage, we looked at phosphorylation of H2AX, a histone variant that is phosphorylated in response to DNA damage [39]. Western blot with an antibody that detects H2AX phosphorylated at Ser139 (γ-H2AX) revealed that plasma treatment of cells induces a dose-dependent increase in γ-H2AX (Figure 2A). Indirect immunofluorescence also revealed foci of γ-H2AX (Figure 2B), which increased in number at higher doses. These data demonstrate that DBD plasma treatment of cells induces a dose-dependent increase in DNA damage.

**Figure 3:**
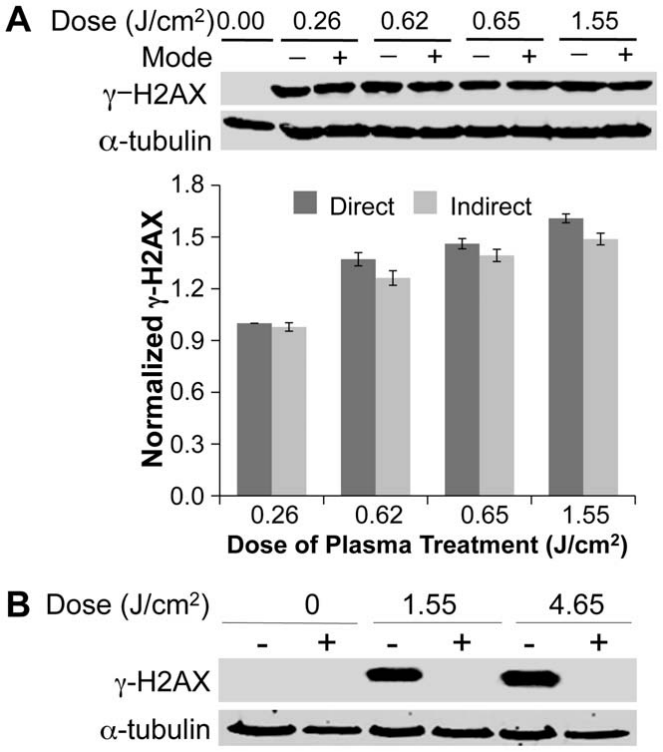
Effects of DBD plasma are mediated by neutral species and not UV generated by plasma in gas phase. (**A**) Cells were subjected to plasma as described earlier (direct, D) or a grounded mesh that filters charged particles was placed between the electrode and the medium (indirect, I). Representative immunoblots with γ-H2AX (upper panel) or α-tubulin (lower panel) are shown. The graphs below the immunoblots show quantification from three independent experiments using the Odyssey Infrared Imaging System (LI-COR Biosciences, Lincoln, NE, USA). The γ-H2AX signal was normalized to the amount of α-tubulin and data are expressed relative to lowest dose that was set at 1.0. (**B**) UV produced in DBD plasma does not induce the observed DNA damage. Cells overlaid with 100 µl of medium were treated with plasma at 1.55 J/cm^2^ and 4.65 J/cm^2^ with (+) and without (−) placing magnesium fluoride (MgF_2_) glass over the cells during treatment. MgF_2_ glass blocks all plasma species except UV from reaching the surface of the medium covering the cells during treatment. Representative immunoblot with γ-H2AX (upper panel) or α-tubulin (lower panel) is shown.

Plasma-induced DNA damage may be initiated by charged and/or neutral species produced by plasma in gas phase. Non-thermal atmospheric pressure DBD plasmas produce relatively long-lived (O_3_, NO, HO_2_, H_2_O_2_) and short-lived (OH, O, electronically excited oxygen O) neutral molecules as well as various charged particles, including ions and electrons [40]. The typical relative concentrations of the various species generated by non-thermal plasma at atmospheric pressure are given in Table 1 [25], [33], [41]. The active species are extremely short-lived at neutral pH and they tend to recombine in the absence of organic substrates with which to react. To determine whether the effects of plasma were due to charged or neutral species, a grounded mesh was used to exclude charged particles (ions and electrons); insertion of a grounded mesh between the plasma source and the media interface (referred to as indirect treatment) did not significantly affect H2AX phosphorylation (Figure 3A), indicating that ions and electrons do not play a significant role and that neutral species produced in the gas phase are responsible for the observed effects. DBD plasma is known to produce UV that is too weak to produce significant effects on living organisms [8], [27], [42]. Blocking all plasma species, except UV, by inserting magnesium fluoride glass during treatment completely blocked the phosphorylation of H2AX after plasma treatment (Figure 3B), further demonstrating that UV does not play a role in plasma-induced DNA damage in mammalian cells.

**Figure 4:**
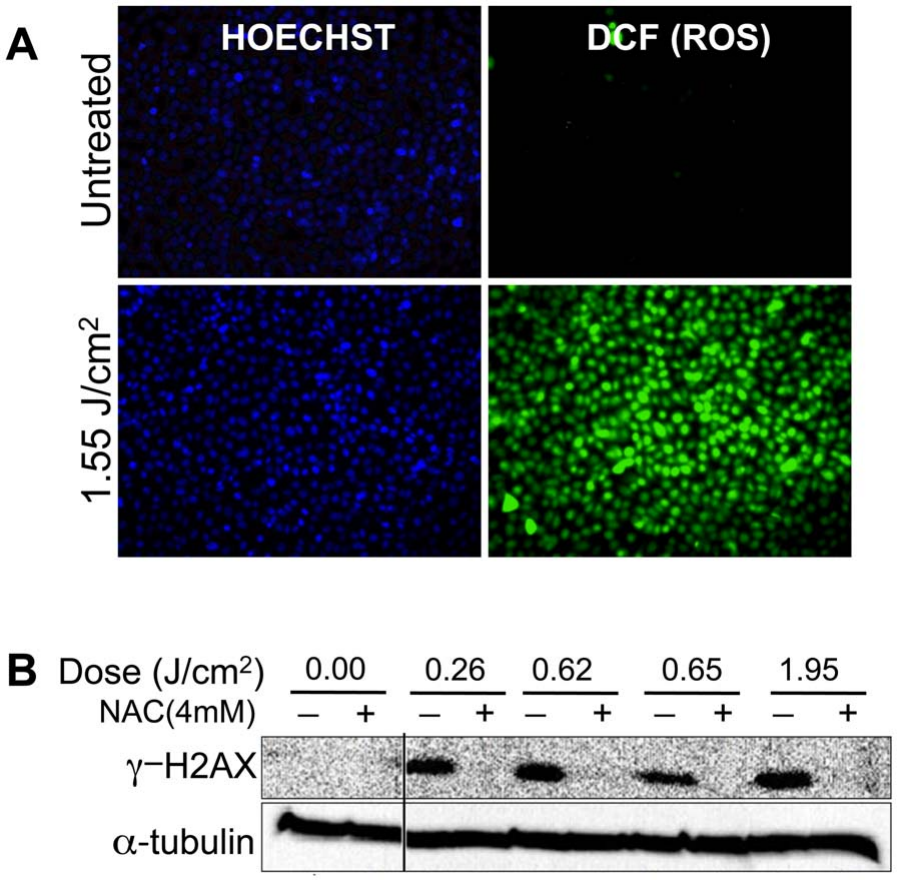
ROS mediate induction of DNA damage by DBD plasma. (**A**) CM-H_2_DCFDA was preloaded into MCF10A cells for 30 min and then the cells were allowed to recover for another 30 min at 37°C. After recovery, MCF10A cells were treated with the indicated dose of DBD plasma. One hour after plasma treatment, intracellular ROS were detected using a fluorescence enabled inverted microscope. (**B**) MCF10A cells were incubated for 2 hours with 4 mM N-acetyl cysteine (NAC) (+), followed by treatment with the indicated dose of DBD plasma. γ-H2AX (top) or α-tubulin (bottom) was detected by immunoblot of cell lysates prepared one hour after plasma treatment.

We next sought to directly test whether the DNA damage induced by DBD plasma is due to the generation of intracellular ROS. We used a common ROS detection dye, CM-H_2_DCFDA, to monitor intracellular ROS levels following plasma treatment. After 60 min incubation, we detected significantly elevated ROS levels in plasma treated MCF10A cells, as compared to the untreated control (Figure 4A). To determine whether DNA damage is induced by the intracellular ROS, we pre-treated cells with the ROS scavenger N-acetyl cysteine (NAC). NAC blocked the induction of γ-H2AX, even at high doses of DBD plasma (Figure 4B), suggesting that the accumulation of DNA damage, as measured by γ-H2AX, is mediated by intracellular ROS.

**Figure 5:**
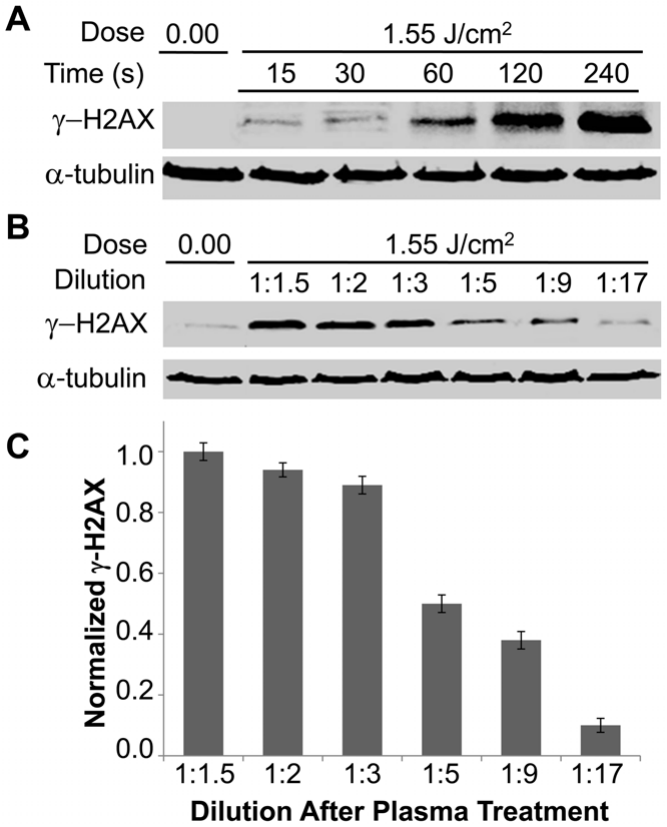
The effects of plasma on cells are dependent on ROS concentration. (**A**) Cells on cover slips overlaid with 100 µl cell culture media were treated with plasma (1.55 J/cm^2^), followed by dilution in 2 ml of media at the indicated holding time after treatment. Cell lysates were collected 1 hour after dilution in 2 ml of media. Immunoblots with γ-H2AX or α-tubulin are shown. (**B**) Medium was separately treated with plasma and then diluted immediately after treatment as indicated. Cells were then exposed to the treated and diluted medium for 1 min, followed by 1-hour incubation in 2 ml of fresh medium. Immunoblots with γ-H2AX or α-tubulin are shown.

Consistent with the result that intracellular ROS mediated plasma induced DNA damage, we found that longer incubation in the original 100 µl of medium, in which cells were treated (before dilution into 2 ml of medium), resulted in higher levels of γ-H2AX (Figure 5A). Additionally, when the treated medium was subjected to different dilutions one minute after treatment, the amount of damage correlated with the dilution, i.e. damage was greater at the lowest dilution (Figure 5B, 5C). These data suggest that the generation of intracellular ROS and the induction of DNA damage are the results of plasma's interaction with the extracellular medium, and that the effects of plasma depend on the concentration of active species in the medium as well as the length of exposure of cells to these active species.

**Figure 6:**
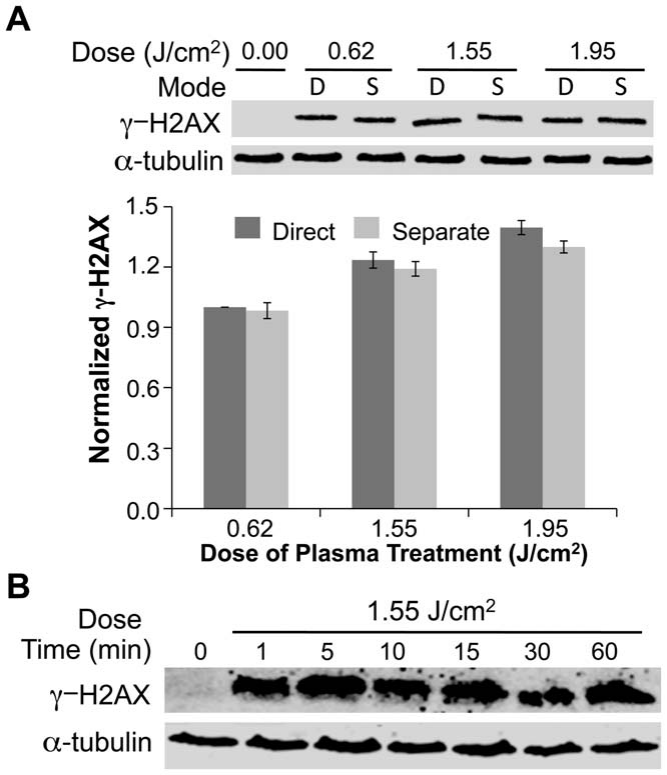
Effects of DBD Plasma are mediated by neutral species generated in the medium. (**A**) Cells were subjected to direct treatment with plasma (D) or to medium (100 µl) that was exposed to plasma and then transferred to the cells (separate, S). (A, B) Representative immunoblots with γ-H2AX (top) or α-tubulin (bottom) are shown. The graphs below the immunoblots show quantification using Odyssey. The γ-H2AX signal was normalized to the amount of α-tubulin. Data are expressed relative to lowest dose, which was set at 1.0. (**B**) Medium (100 µl separated treatment) was subjected to plasma and transferred to cells after holding for 1 to 60 min. After 1-minute incubation with cells, cover slips with treated medium and cells were transferred to a dish with 2 ml of medium.

**Figure 7:**
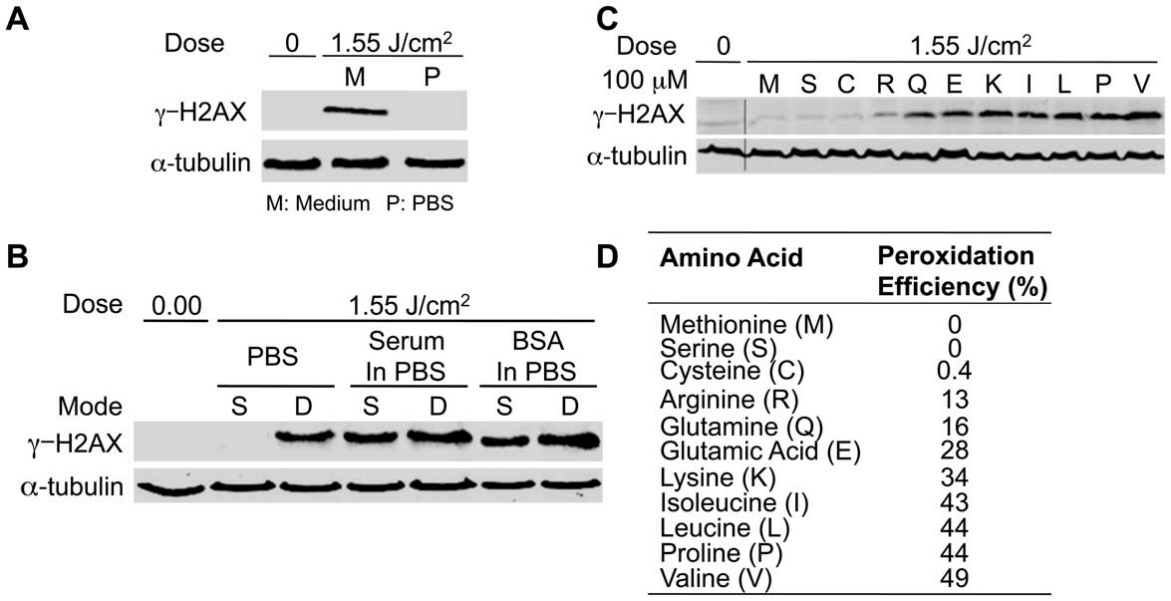
Amino acid hydroperoxides produced by plasma treatment of organic medium induce DNA damage. (**A**) Cells were treated with 100 µl of medium or PBS that was separately exposed to 1.55 J/cm^2^ plasma. (**B**) 100 µl of PBS, medium without serum, or PBS with 100 µg/ml BSA were treated with plasma (1.55 J/cm^2^) and immediately added to cells on a coverslip (separated, S). Cells overlaid with 100 µl of the indicated solution were treated with plasma (1.55 J/cm^2^) (direct, D). (**C**) Solutions containing the indicated amino acid (100 µM) were separately treated with plasma and then added to MCF10A cells. (**A, B, C**) After 1-minute incubation, cells on cover slips were diluted in 2 ml medium, followed by lysis and Western blot for γ-H2AX and α-tubulin. (**D**) Peroxidation efficiency of various amino acid components of cell culture medium when treated with IR [43]. For each amino acid, the amount of DNA damage induced is proportional to the peroxidation efficiency.

To further establish that the effects of DBD plasma are due to modification of the cell medium by the plasma treatment as opposed to a direct effect on the cells, medium was treated separately and added to cells. Medium (100 µl) on a coverslip (without cells) was treated with DBD plasma and then transferred to a fresh coverslip with cells, which we have termed ‘separated treatment’. The effect of medium separately treated with DBD plasma and added to cells was not significantly different from the effect of direct treatment of cells overlaid with medium (Figure 6A). To begin addressing which component of the medium is affected by the treatment, we assessed the stability of the separately treated medium. Separately treated medium was held for increasing times before being added to cells. Induction of DNA damage by the treated medium was not reduced by holding the medium up to one hour prior to adding it to cells (Figure 6B), suggesting that the active species formed in the medium are relatively stable. To identify the active components, we compared the effect of separated treatment of complete medium vs. inorganic phosphate buffered saline (PBS). We observed no DNA damage in cells exposed to separately treated PBS (Figure 7A), whereas separately treated medium induced DNA damage as anticipated. This suggests that stable organic components in the medium, such as organic peroxides [43] mediate the observed effects.

Cell culture medium is composed of amino acids, glucose, vitamins, growth factors and inorganic salts, as well as serum. Gebicki et al. have shown that γ-radiation (IR) induces formation of amino acid and protein hydroperoxides in aqueous solutions containing BSA or individual amino acids [43]. Equivalent levels of H2AX phosphorylation were induced in cells subjected to separately treated serum-containing medium, serum-free medium or PBS with BSA, but not PBS alone (Figure 7B), suggesting that amino acid peroxidation may be involved. Peroxidation efficiency is widely variable among different amino acids [43]. To determine whether the observed results were related to the peroxidation efficiency of organic components in cell culture medium, 11 different amino acids with a range of peroxidation efficiencies were dissolved individually in PBS and separately treated with plasma and then added to cells. As shown in Figure 7C, phosphorylation of H2AX was directly proportional to the peroxidation efficiency of the amino acids, with valine producing the most significant level of damage and serine and methionine producing no detectable DNA damage (Figure 7D). There is a direct correlation between the peroxidation efficiency of 11 different amino acids and the level of DNA damage, providing strong support for the hypothesis that organic peroxides are produced in the plasma treated medium and are responsible for the observed effects on DNA.

**Figure 8:**
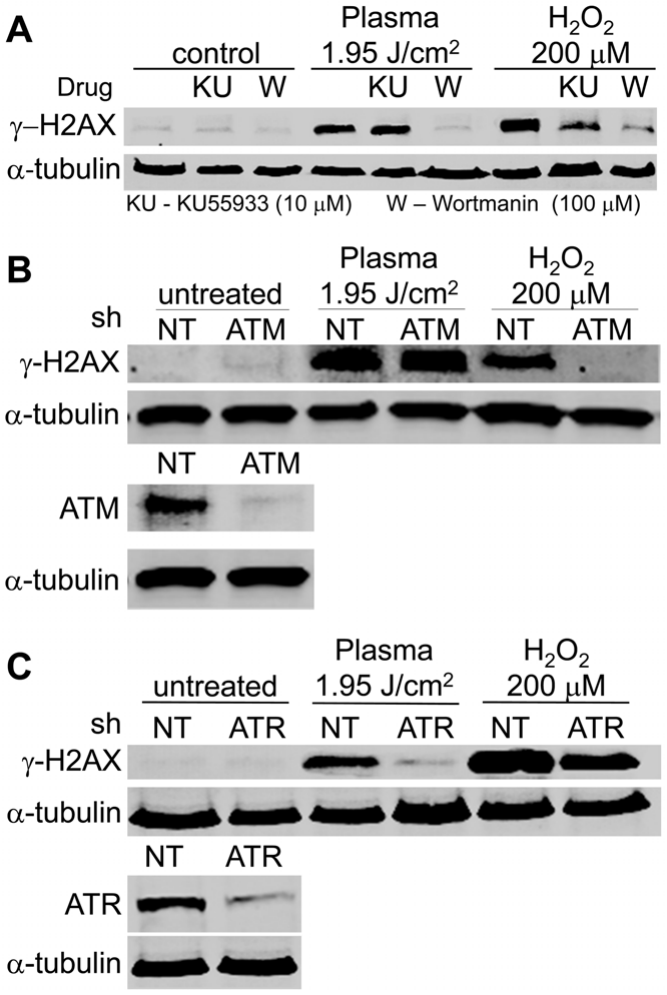
ATR dependence of non-thermal plasma induced phosphorylation of H2AX. (**A**) Immunoblot of γ-H2AX (top), and α-tubulin (bottom) from MCF10As exposed to non-thermal plasma at a dose of 1.95 J/cm^2^ or 200 µM H_2_O_2_ in the presence (+) or absence (−) of 100 µmol/L Wortmannin (Wort.) or 10 µmol/L KU55933 (KU). (**B**) MCF10As were depleted of endogenous ATM by shRNA for 72 hours (bottom, immunoblot of ATM after ATM or non-targeting (NT) shRNA). Cells were then plated on glass cover slips and exposed to DBD plasma at a dose of 1.95 J/cm^2^ or 200 µM H_2_O_2_. (**C**) MCF10As depleted of endogenous ATR by shRNA for 72 hours (bottom, immunoblot of ATR after ATR or non-targeting (NT) shRNA). (**B,C**) After knockdown, cells were plated on glass cover slips for 24 h followed by exposure to non-thermal plasma at a dose of 1.95 J/cm^2^ or 200 µM H_2_O_2_. After one-hour incubation, lysates were prepared and resolved by SDS-PAGE and representative immunoblots with antibody to γ-H2AX (top) or α-tubulin (bottom) are shown.

To characterize the DNA damage pathway activated by DBD plasma treatment, we next sought to identify the kinase that phosphorylates H2AX in response to DBD plasma treatment. As discussed, ATM, ATR and DNA-PK can phosphorylate H2AX on Ser139. Phosphorylation of H2AX in response to plasma (as well as H_2_O_2_) was markedly reduced in cells pretreated with 100 µM Wortmannin (Figure 8A), which at 100 µM inhibits ATM, ATR, and DNA-PK [44]. In contrast, one hour pretreatment with 10 µM KU55933, an ATM specific inhibitor [45], did not significantly reduce the phosphorylation of H2AX in response to plasma treatment, whereas it significantly reduced it in response to H_2_O_2_ (Figure 8A). These findings indicate that ATR and/or DNA-PK is required for the phosphorylation of H2AX at Ser139 in response to non-thermal plasma, although they do not rule out that other kinases may be activated.

To more directly assess the role of ATM and/or ATR, shRNAs were utilized. ATM shRNA effectively reduced levels of ATM and blocked phosphorylation of H2AX in response to hydrogen peroxide, but did not significantly affect the phosphorylation of H2AX induced by plasma treatment (1.95 J/cm^2^) as compared to non-targeting shRNA (Figure 8B). These findings confirm the results with inhibitors and indicate that ATM is not the primary mediator of H2AX phosphorylation in response to plasma treatment. Depletion of ATR by shRNA reduced phosphorylation of H2AX in response to plasma treatment by 92% relative to non-targeting shRNA and 40% in response to hydrogen peroxide (Figure 8C). Taken together, our findings demonstrate that non-thermal plasma treatment activates ATR.

**Figure 9:**
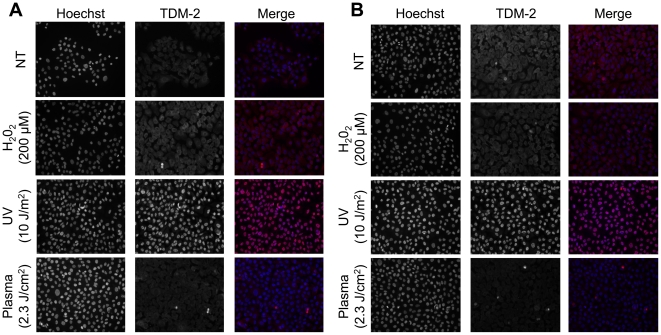
Plasma treatment does not induce the formation of thymine dimers. MCF10A cells grown on glass coverslips were incubated with (**A**) or without (**B**) 4 mM NAC for 2 h, followed by treatment with direct plasma (2.3 J/cm^2^), UV (10 J/m^2^), H_2_O_2_ (200 µM), or a no treatment (NT) control. Cells were allowed to recover for 1 h, then fixed and immunostained with TDM-2 primary antibody (kindly provided by Dr. Toshio Mori at the Nara Medical University, Kashihara, Nara, Japan) and Alexa Fluor 594 anti-mouse secondary antibody to detect cyclobutane pyrimidine dimers.

Activation of ATR by UV is through the formation of thymine dimers resulting in replication fork collapse [46]. To determine whether plasma treatment resulted in formation of thymine dimers, immunofluorescence with an antibody that detects cyclobutane pyrimidine dimers (TDM-2) was performed on cells after treatment with UV or plasma. As shown in Figure 9A, all of the cells treated with UV showed the presence of cyclobutane pyrimidine dimers, whereas there was no evidence of thymine dimers in cells treated with plasma. Pretreatment of cells with NAC did not prevent formation of bulky adducts/thymine dimers in UV treated cells (Figure 9B), further supporting that the effects of plasma are different from UV.

## Discussion

Based on the myriad of potential clinical applications of non-thermal plasma [26] and the lack of information about the molecular basis of the effects of plasma on mammalian cells, this study has addressed the interaction of plasma with cells at a molecular level. The present study demonstrates that non-thermal plasma produces dose-dependent effects that range from increased cell proliferation to apoptosis; these effects are the result of the production of intracellular ROS.

Cell death in response to non-thermal plasma treatment in the dose range examined is primarily though induction of apoptosis, which is an important therapeutic consideration. Apoptotic cells are broken up into apoptotic bodies, which are engulfed by neighboring cells, leading to cell death without significant inflammatory response [47], [48]. Controlled delivery of non-thermal plasma may provide a means to kill benign and malignant lesions in a defined area, without significant necrosis and subsequent inflammation. Delivery can be achieved by direct treatment of tissue surfaces or application of defined medium treated with plasma.

Plasma-induced DNA damage is likely initiated by neutral species and not charged species produced by plasma in gas phase. Use of a grounded mesh to exclude charged particles (ions and electrons) did not significantly affect H2AX phosphorylation. Further, blocking of all plasma species, except UV (by a magnesium fluoride glass placed over the cells during treatment) completely blocked the phosphorylation of H2AX after plasma treatment, further demonstrating that UV does not play a role in plasma-induced DNA damage in mammalian cells. Moreover, the results of the experiments comparing direct treatment with separated plasma treatment further rule out any effect of plasma resulting from UV, temperature or electric field.

Mammalian cells undergo DNA damage as a result of ROS generated from endogenous and exogenous sources. The intracellular ROS scavenger, NAC, completely blocked phosphorylation of H2AX after non-thermal plasma treatment of MCF10A cells, indicating that the induction of DNA damage is mediated through the formation of intracellular ROS. The role of ROS is further supported by direct measurement of intracellular ROS and by the formation of lipid peroxidation products (Figure S2A, Methods S1), specifically malondialdehyde (MDA); however, plasma-induced lipid peroxidation does not lead to phosphorylation of H2AX (Figure S2B).

We have provided several lines of evidence that the interaction of plasma with organic components of cells and media is responsible for the induction of DNA damage. First, cells exposed to PBS that was separately treated with plasma did not exhibit DNA damage, whereas cells exposed to medium or a solution of specific amino acids did undergo DNA damage and the media remains active over extended periods of time, which would not be the case with inorganic peroxides (e.g. H_2_O_2_), and is consistent with published reports on organic peroxides [43]. The formation of intracellular ROS as a result of plasma treatment can result from formation of organic peroxides in the medium; in support of this notion, there is a direct correlation between the peroxidation efficiency of 11 different amino acids and the amount of DNA damage induced in cells exposed to these amino acid solutions after plasma treatment. Taken together, these data strongly support the conclusion that the effects of DBD plasma are mediated by the organic peroxides formed in the medium, although formation of organic peroxides in plasma-treated medium should be directly measured. It should be noted that cells in PBS directly treated with plasma exhibit DNA damage, in contrast to cells exposed to separately treated PBS which do not. We presume that the damage in cells after treatment in PBS is the result of short-lived species interacting directly with oxidizable organic substrates (including DNA) in cells. That the separated treatment of PBS does not induce damage suggests that in absence of organic substrates, the ROS that are generated are short-lived probably due to their recombination and consequently are no longer active when added to cells after the separated treatment. The only possible explanation for the observed effects of direct plasma treatment in PBS is that ROS coming directly from plasma interact with organic substrates in cells producing stable ROS. This is supported by our data demonstrating that the effects of separated or direct treatment are blocked by an ROS scavenger.

Complex molecular networks that rapidly sense and repair DNA damage have evolved to maintain genomic stability and ensure cell survival [49]. ATM, ATR and DNA-PK [44] can phosphorylate H2AX on Ser139 over a large region of chromatin surrounding a DSB or a stalled replication fork [50], [51]. In this study, we have shown that phosphorylation of H2AX after plasma treatment of MCF10A cells is primarily through ATR, in contrast to the ATM-dependent phosphorylation of H2AX after treatment of cells with IR or hydrogen peroxide. These results suggested that plasma treatment may lead to formation of bulky adducts; however, as shown, plasma treatment does not lead to formation of bulky adducts/thymine dimers in MCF10A cells. Unlike IR or H_2_O_2_, non-thermal plasma treatment may lead to formation of stalled replication forks [52] to activate ATR; however, unlike UV, the activation of ATR by plasma does not involve formation of thymine dimers and it is mediated by formation of ROS. Further studies are needed to determine if plasma induces formation of regions of single stranded DNA or interstrand cross-links which would activate ATR.

Understanding the mechanism underlying the effects of plasma is essential in applying it to clinical use. We have shown here that DBD plasma generates active oxygen species in gas phase, which can react with organic components, such as serum or amino acids, to produce long-lived reactive species, mostly likely amino acid and protein hydroperoxides. The amount of intracellular ROS produced by plasma can be controlled by varying the frequency and voltage waveform, allowing for the fine-tuning of the therapeutic effect, from stimulating cell proliferation to inducing apoptosis. Generation of organic peroxides in solution may provide an alternative means of administration. Future work will involve investigating the mechanism by which peroxidized amino acids are processed by the cell. Current studies are directed at establishing whether the effects of plasma are through the uptake of amino acid hydroperoxides by active transport mechanisms in cells or through ROS signaling and determining the nature of the DNA damage. Devices that deliver DBD plasma can be designed to accommodate a wide range of clinical applications, from surface to endoscopic administration. Possible clinical applications of DBD plasma include wound sterilization, potential enhancement of healing, controlled ablation of tissue, and selective targeting of benign or malignant lesions.

## Supporting Information

Figure S1 Schematic of the plasma treatment setup.
(TIF)

Figure S2 DBD plasma-induced lipid peroxidation is not responsible for the observed DNA damage. (**A**) Cells overlaid with 100 μl of medium were treated with plasma at 1.55 J/cm^2^ with (+) and without (-) pre-incubating for 2 h with diphenyl phenyl enediamine (DPPD, Sigma-Aldrich, St. Louis, MO, USA) a lipophilic antioxidant which blocks lipid peroxidation. (**B**) Cells overlaid with 100 μl of medium were treated with plasma (1.55 J/cm^2^) with (+) and without (−) pre-incubating with DPPD. 1 h after plasma treatment cells were lysed and immunoblots were prepared to detect DNA damage by looking at γ-H2AX signal. Representative immunoblot for γ-H2AX (upper panel) or α-tubulin (lower panel) with quantification below it is shown. The γ-H2AX signal was normalized to the amount of α-tubulin. Data are expressed relative to the amount of γ-H2AX in plasma-treated sample without DPPD, which was set to 1.0.
(TIF)

Methods S1 Supplementary methods.
(DOC)

## References

[1] Vargo JJ (2004). Clinical applications of the argon plasma coagulator.. Gastrointest Endosc.

[2] Sladek REJ, Stoffels E (2005). Deactivation of Escherichia coli by the plasma needle.. Journal of Physics D: Applied Physics.

[3] Goree J, Bin l, Drake D, Stoffels E (2006). Killing of S. mutans Bacteria Using a Plasma Needle at Atmospheric Pressure.. IEEE Transactions on Plasma Science.

[4] Stoffels E, Kieft AIE, Sladek AREJ, Zandvoort V, Slaaf DW, d'Agostino R, Favia P, Kawai Y, Ikegami H, Sato N (2008). Cold gas plasma in biology and medicine.. Advanced Plasma Technology.

[5] Stoffels E (2006). Gas plasmas in biology and medicine.. Journal of Physics D: Applied Physics.

[6] Laroussi M, Tendero C, Lu X, Alla S, Hynes WL (2006). Inactivation of Bacteria by the Plasma Pencil.. Plasma Processes and Polymers.

[7] Laroussi M, Mendis DA, Rosenberg M (2003). Plasma interaction with microbes.. New Journal of Physics.

[8] Laroussi M, Leipold F (2004). Evaluation of the roles of reactive species, heat, and UV radiation in the inactivation of bacterial cells by air plasmas at atmospheric pressure.. International Journal of Mass Spectrometry.

[9] Laroussi M, Alexeff I, Kang WL (2000). Biological decontamination by nonthermal plasmas.. Plasma Science, IEEE Transactions on.

[10] Laroussi M (2005). Low Temperature Plasma-Based Sterilization: Overview and State-of-the-Art.. Plasma Processes and Polymers.

[11] Fridman G, Peddinghaus M, Ayan H, Fridman A, Balasubramanian M (2006). Blood Coagulation and Living Tissue Sterilization by Floating-Electrode Dielectric Barrier Discharge in Air *Plasma Chemistry and Plasma Processing*.

[12] Kieft IE, Kurdi M, Stoffels E (2006). Reattachment and Apoptosis After Plasma-Needle Treatment of Cultured Cells.. Plasma Science, IEEE Transactions on.

[13] Kieft IE, Darios D, Roks AJM, Stoffels E (2005). Plasma treatment of mammalian vascular cells: a quantitative description.. Plasma Science, IEEE Transactions on.

[14] Kalghatgi S, Fridman G, Cooper M, Nagaraj G, Peddinghaus M (2007). Mechanism of Blood Coagulation by Nonthermal Atmospheric Pressure Dielectric Barrier Discharge Plasma.. Plasma Science, IEEE Transactions on.

[15] Kalghatgi S, Friedman G, Fridman A, Clyne AM (2010). Endothelial Cell Proliferation is Enhanced by Low Dose Non-Thermal Plasma Through Fibroblast Growth Factor-2 Release.. Ann Biomed Eng.

[16] Coulombe S, Léveillé V, Yonson S, Leask RL (2006). Miniature atmospheric pressure glow discharge torch (APGD-t) for local biomedical applications.. Pure and Applied Chemistry.

[17] Leduc M, Guay D, Leask RL, Coulombe S (2009). Cell permeabilization using a non-thermal plasma.. New Journal of Physics.

[18] Jiang C, Vernier PT, Chen MT, Wu YH, Wang LL (2008). Low Energy Nanosecond Pulsed Plasma Sterilization for Endodontic Applications.

[19] Xinpei L, Yinguang C, Ping Y, Qing X, Zilan X (2009). An *RC* Plasma Device for Sterilization of Root Canal of Teeth.. Plasma Science, IEEE Transactions on.

[20] Sladek REJ, Stoffels E, Walraven R, Tielbeek PJA, Koolhoven RA (2004). Plasma treatment of dental cavities: a feasibility study.. Plasma Science, IEEE Transactions on.

[21] Shekhter AB, Serezhenkov VA, Rudenko TG, Pekshev AV (2005). Beneficial effect of gaseous nitric oxide on the healing of skin wounds.. Nitric Oxide: Biology and Chemistry.

[22] Fridman A, Chirokov A, Gutsol A (2005). Non-thermal atmospheric pressure discharges.. Journal of Physics D: Applied Physics.

[23] Siemens CW (1862). On the Electrical Tests Employed During the Construction of the Malta and Alexandria Telegraph, and on Insulating and Protecting Submarine Cables *Journal of the Franklin Institute*.

[24] Eliasson B, Egli W, Kogelschatz U (1994). Modelling of dielectric barrier discharge chemistry.. Pure and Applied Chemistry.

[25] Fridman A, Kennedy LA (2004). Plasma Physics and Engineering: Routledge, USA..

[26] Fridman G, Friedman G, Gutsol A, Shekhter AB, Vasilets VN (2008). Applied Plasma Medicine.. Plasma Processes and Polymers.

[27] Fridman G, Brooks A, Balasubramanian, Fridman A, Gutsol A (2007). Comparison of Direct and Indirect Effects of Non-Thermal Atmospheric-Pressure Plasma on Bacteria.. Plasma Processes and Polymers.

[28] Weng C-C, Wu Y-T, Liao J-D, Kao C-Y, Chao C-C (2009). Inactivation of bacteria by a mixed argon and oxygen micro-plasma as a function of exposure time.. International Journal of Radiation Biology.

[29] Olofsson BA, Kelly CM, Kim J, Hornsby SM, Azizkhan-Clifford J (2007). Phosphorylation of Sp1 in response to DNA damage by ataxia telangiectasia-mutated kinase.. Mol Cancer Res.

[30] Lehnert BE, Iyer R (2002). Exposure to low-level chemicals and ionizing radiation: reactive oxygen species and cellular pathways.. Hum Exp Toxicol.

[31] Rogakou E, Boon C, Redon C, Bonner W (1999). Megabase chromatin domains involved in DNA double-strand breaks in vivo.. J Cell Biol.

[32] Ayan H, Fridman G, Staack D, Gutsol AF, Vasilets VN (2009). Heating Effect of Dielectric Barrier Discharges for Direct Medical Treatment.. Plasma Science, IEEE Transactions on.

[33] Fridman A (2008). Plasma Chemistry: Cambridge University Press..

[34] Kogelschatz U, Becker KH, Schoenbach KH, Barker RJ (2004). Non-Equilibrium Air Plasmas at Atmospheric Pressure: Taylor & Francis..

[35] Fridman A (2008). Plasma Biology and Plasma Medicine..

[36] Mirzoeva OK, Petrini JH (2001). DNA damage-dependent nuclear dynamics of the Mre11 complex.. Mol Cell Biol.

[37] Fitch ME, Cross IV, Ford JM (2003). p53 responsive nucleotide excision repair gene products p48 and XPC, but not p53, localize to sites of UV-irradiation-induced DNA damage, in vivo.. Carcinogenesis.

[38] Bomgarden RD, Lupardus PJ, Soni DV, Yee MC, Ford JM (2006). Opposing effects of the UV lesion repair protein XPA and UV bypass polymerase eta on ATR checkpoint signaling.. EMBO J.

[39] Rogakou E, Pilch D, Orr A, Ivanova V, Bonner W (1998). DNA double-stranded breaks induce histone H2AX phosphorylation on serine 139.. J Biol Chem.

[40] Fridman A (2008). Plasma Biology and Plasma Medicine.. Plasma Chemistry.

[41] Fridman A, Chirokov A, Gutsol A (2005). Non-thermal atmospheric pressure discharges.. Journal of Physics D: Applied Physics.

[42] Dobrynin D, Fridman G, Friedman G, Fridman A (2009). Physical and biological mechanisms of direct plasma interaction with living tissue..

[43] Gebicki S, Gebicki JM (1993). Formation of peroxides in amino acids and proteins exposed to oxygen free radicals.. Biochem J.

[44] Abraham RT (2004). PI 3-kinase related kinases: 'Big' players in stress-induced signaling pathways.. DNA Repair.

[45] Hickson I, Zhao Y, Richardson CJ, Green SJ, Martin NMB (2004). Identification and Characterization of a Novel and Specific Inhibitor of the Ataxia-Telangiectasia Mutated Kinase ATM.. Cancer Res.

[46] Ward IM, Minn K, Chen J (2004). UV-induced Ataxia-telangiectasia-mutated and Rad3-related (ATR) Activation Requires Replication Stress.. Journal of Biological Chemistry.

[47] Fiers W, Beyaert R, Declercq W, Vandenabeele P (1999). More than one way to die: apoptosis, necrosis and reactive oxygen damage.. Oncogene.

[48] Majno G, Joris I (1995). Apoptosis, Oncosis, and Necrosis - an Overview of Cell-Death.. American Journal of Pathology.

[49] Shiloh Y (2006). The ATM-mediated DNA-damage response: taking shape.. Trends Biochem Sci.

[50] Rogakou EP, Boon C, Redon C, Bonner WM (1999). Megabase chromatin domains involved in DNA double-strand breaks in vivo.. J Cell Biol.

[51] Rogakou EP, Pilch DR, Orr AH, Ivanova VS, Bonner WM (1998). DNA doublestranded breaks induce histone H2AX phosphorylation on serine 139.. Journal of biological chemistry.

[52] Ward IM, Chen J (2001). Histone H2AX Is Phosphorylated in an ATR-dependent Manner in Response to Replicational Stress.. The Journal of Biological Chemistry.

