# Supplementary figures and images for: Effects of Non-Thermal Plasma on Mammalian Cells
Source: PLoS One. 2011 Jan 21;6(1):e16270. doi: 10.1371/journal.pone.0016270 (PMC3025030; doi:10.1371/journal.pone.0016270)

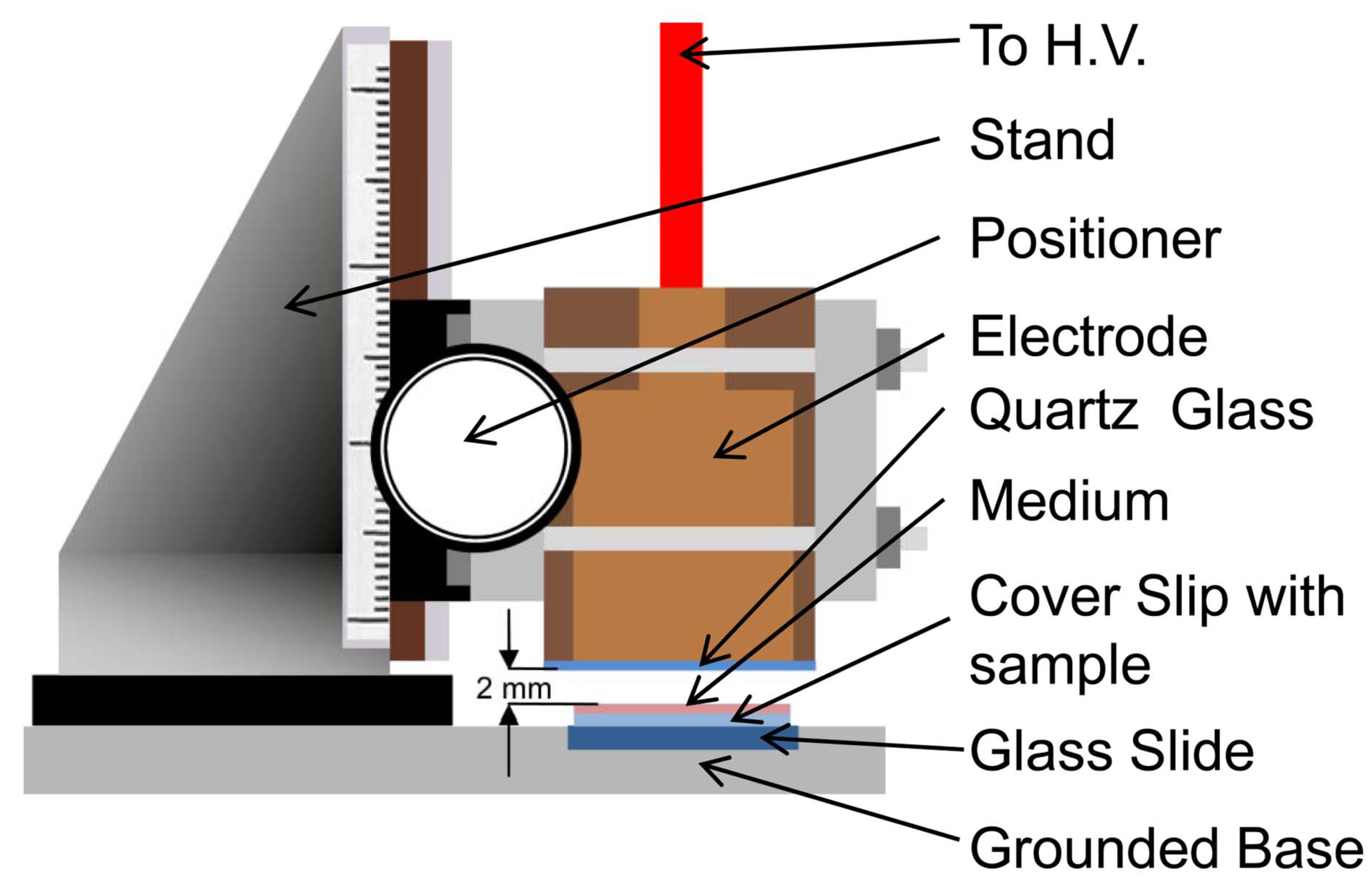

Supplement: Figure S1 — Schematic of the plasma treatment setup. (TIF) [file pone.0016270.s001.tif]

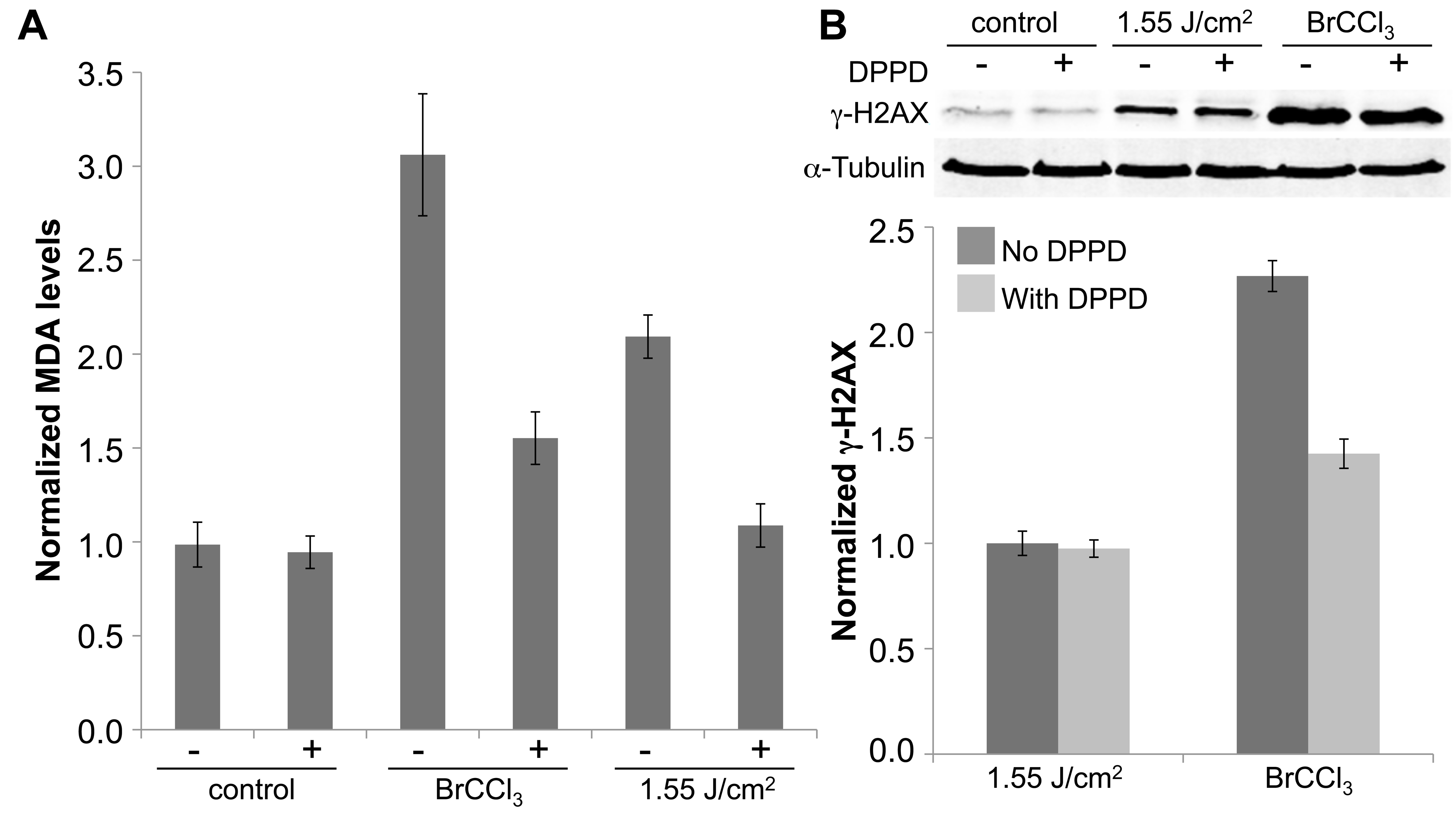

Supplement: Figure S2 — DBD plasma-induced lipid peroxidation is not responsible for the observed DNA damage. (A) Cells overlaid with 100 μl of medium were treated with plasma at 1.55 J/cm2 with (+) and without (-) pre-incubating for 2 h with diphenyl phenyl enediamine (DPPD, Sigma-Aldrich, St. Louis, MO, USA) a lipophilic antioxidant which blocks lipid peroxidation. (B) Cells overlaid with 100 μl of medium were treated with plasma (1.55 J/cm2) with (+) and without (−) pre-incubating with DPPD. 1 h after plasma treatment cells were lysed and immunoblots were prepared to detect DNA damage by looking at γ-H2AX signal. Representative immunoblot for γ-H2AX (upper panel) or α-tubulin (lower panel) with quantification below it is shown. The γ-H2AX signal was normalized to the amount of α-tubulin. Data are expressed relative to the amount of γ-H2AX in plasma-treated sample without DPPD, which was set to 1.0. (TIF) [file pone.0016270.s002.tif]
