# Supplementary material for: Effects of Non-Thermal Plasma on Mammalian Cells
Source: PLoS One. 2011 Jan 21;6(1):e16270. doi: 10.1371/journal.pone.0016270 (PMC3025030; doi:10.1371/journal.pone.0016270)
Supplement: Methods S1 — Supplementary methods. (DOC) [file pone.0016270.s003.doc]

**Lipid Peroxidation**

Malondialdehyde-thiobarbituric acid (MDA-TBA) levels were used as a measure of lipid peroxidation after plasma treatment of mammalian cells. Bromotrichloromethane (BrCCl3, Sigma-Aldrich, St. Louis, MO, USA) was used as a known inducer of lipid peroxidation. Cells were held after plasma treatment for 1 min before addition of butylated hydroxtoluene (BHT) to prevent further lipid peroxidation while carrying out the assay. The cells were scrapped with a rubber policeman and homogenized at 4°C. Whole lysates were used to measure the level of MDA following the manufacturer’s protocol (OxiSelect^TM^ TBARS Assay kit, Cell BioLabs, San Diego, CA, USA). The TBA test was carried out under acidic (pH 3.5) conditions with a colorimetric 96-well microplate assay, and the level of TBARS was expressed relative to the response of the assay to malondialdehyde (MDA) using a plate reader at 532 nm.
